# Supplementary material for: Explicit and Implicit Measures of Black Cat Bias in Cat and Dog People
Source: Animals (Basel). 2024 Nov 22;14(23):3372. doi: 10.3390/ani14233372 (PMC11640230; doi:10.3390/ani14233372)
Supplement: Supplementary file 1 [file animals-14-03372-s001.zip › animals-3281238-supplementary.pdf]

Table S1. Descriptive Statistics for the Belief in Paranormal Phenomena Questionnaire for Study 1

	**Mean**	**Std. Deviation**
Religious Beliefs	1.93	1.29
PSI	1.17	0.96
Witchcraft	1.54	1.09
Superstition	0.59	0.78
Spiritualism	1.61	1.16
Extraordinary Life Forms	1.11	0.98
Precognition	1.96	1.13

Note. N = 114. The maximum possible value was 4. Larger means indicate higher amounts of the belief.

Table S2. Descriptive Statistics for the Belief in Paranormal Phenomena Questionnaire for Study 2, N = 114

	**Time 1**		**Time 2**	
	**Mean**	**Std. Deviation**	**Mean**	**Std. Deviation**
Religious Beliefs	3.07	0.72	3.09	0.62
PSI	1.40	0.27	1.53	0.16
Witchcraft	1.05	0.70	1.21	0.80
Superstition	1.24	0.89	1.23	0.89
Spiritualism	1.72	0.67	1.83	0.72
Extraordinary Life Forms	0.96	0.84	0.93	0.87
Precognition	2.15	0.87	2.16	0.91

Note. N = 37 – the participants who completed the study at both time 1 and 2.
